# Effects of Preformed Composition and Pore Size on Microstructure and Properties of SiC*_f_*/SiC Composites via Reactive Melt Infiltration

**DOI:** 10.3390/ma17235765

**Published:** 2024-11-25

**Authors:** Haifeng Nie, Pingzhan Si, Quanxing Ren, Ziqiang Yin, Tihao Cao, Zhengren Huang, Qing Huang, Yinsheng Li

**Affiliations:** 1 College of Materials Science and Engineering, China Jiliang University, Hangzhou 310018, China; niehaifeng@nimte.ac.cn (H.N.); pzsi@cjlu.edu.cn (P.S.); 2 Zhejiang Key Laboratory of Data-Driven High-Safety Energy Materials and Applications, Ningbo Key Laboratory of Special Energy Materials and Chemistry, Ningbo Institute of Materials Technology and Engineering, Chinese Academy of Sciences, Ningbo 315201, China; renquanxing@nimte.ac.cn (Q.R.); yinziqiang@nimte.ac.cn (Z.Y.); zhrhuang@nimte.ac.cn (Z.H.); huangqing@nimte.ac.cn (Q.H.); 3 Qianwan Institute of CNITECH, Ningbo 315336, China; 4 Advanced Energy Science and Technology Guangdong Laboratory, Huizhou 516003, China; 5 University of Chinese Academy of Sciences, Beijing 100049, China

**Keywords:** SiC*_f_*/SiC composites, reactive melt infiltration, preformed composition, pore size, properties

## Abstract

This study investigated the influence of preformed composition and pore size on the microstructure and properties of SiC*_f_*/SiC composites fabricated via reactive melt infiltration (RMI). The process began with the impregnation of SiC fiber cloth with phenolic resin, followed by lamination and pyrolysis. Subsequent steps included further impregnations with phenolic resin, SiC slurry, and carbon black slurry, each followed by additional pyrolysis. This process resulted in three types of preforms, designated as PP, PS, and PC. These preforms exhibited a multimodal distribution of pore size, with peak pore diameters around 5 μm for PP, ranging from 200 nm to 4 μm for PS, and approximately 150 nm for PC. The preforms were then subjected to molten silicon infiltration at 1600 °C under vacuum for 1 h to create SiC*_f_*/SiC composites. The PP preform contained only pyrolytic carbon, leading to a composite with high closed porosity and unreacted carbon, resulting in poor mechanical properties. The PS preform, which was impregnated with SiC particles, displayed an optimized pore size distribution but retained significant amounts of residual silicon and carbon in the final composite. In contrast, the PC preform featured both an ideal pore size distribution and an adequate amount of carbon, achieving high density and low porosity with reduced residual phases in the final composite. This optimization led to a flexural strength of 152.4 ± 15.4 MPa, an elastic modulus of about 181.1 ± 0.1 GPa, and a thermal conductivity of 27.7 W/mK in the SiC*_f_*/SiC composites product. These findings underscore the importance of preform optimization in enhancing the performance of SiC*_f_*/SiC composites, potentially paving the way for more reliable nuclear fuel cladding solutions.

## 1. Introduction 

SiC fiber-reinforced SiC matrix composites (SiC*_f_*/SiC) possess significant potential for applications in aerospace, defense, and nuclear industries due to their exceptional properties, including their lightweight design, high strength, high toughness, corrosion resistance, high-temperature resistance, radiation resistance, and oxidation resistance [1,2,3,4,5]. Furthermore, SiC*_f_*/SiC ceramic matrix composites typically do not experience catastrophic failure due to their non-brittle fracture characteristics, which are less sensitive to cracks [1,2,6]. Benefiting from these excellent properties, SiC*_f_*/SiC composites have become highly promising candidates for accident-tolerant nuclear fuel cladding. They could effectively prevent disasters like the Fukushima nuclear accident, which was primarily caused by the zirconium–water reaction of zirconium alloy cladding tubes at high temperatures, resulting in the production of large amounts of hydrogen and triggering a hydrogen explosion [7]. Compared to zirconium alloys, SiC*_f_*/SiC composites offer several advantages: (1) superior temperature resistance, allowing for long-term use at 800 °C and short-term exposure to temperatures exceeding 1200 °C; (2) a neutron absorption cross-section that is 15% lower than that of zirconium alloys; and (3) enhanced mechanical properties, including lightweight construction, high strength, high toughness, and high hardness [6,8]. These advantages have enabled SiC*_f_*/SiC composites to be an ideal structural material in advanced nuclear energy systems.

SiC*_f_*/SiC composites are primarily fabricated through various techniques, such as the chemical vapor infiltration (CVI) [9,10,11], polymer infiltration and pyrolysis (PIP) [12,13,14], reactive melt infiltration (RMI) [15,16,17], and nano-infiltration transient eutectic (NITE) [18,19,20] methods. Among these, the RMI approach yields SiC*_f_*/SiC composites with exceptionally low porosity and high density, which facilitate net shaping during material preparation. This capability allows for the fabrication of composite materials with intricate geometries. The RMI process involves the creation of a porous SiC*_f_*/C preform, followed by the formation of a SiC ceramic matrix to produce the SiC*_f_*/SiC composites. Typically, the porous SiC*_f_*/C preform is generated by introducing carbon into the woven SiC fiber structure using CVI or PIP processes, utilizing carbon sources like carbon particles or resin. The SiC*_f_*/SiC composites are then formed through reactions between the carbon in the SiC*_f_*/C preform and molten silicon via liquid-phase silicon infiltration (LSI) or vapor-phase silicon infiltration (VSI). Although SiC*_f_*/SiC composites produced by the RMI method exhibit extremely low porosity, high density, and facilitate net shaping during material preparation, controlling the silicon and carbon contents during RMI presents challenges. Indeed, controlling the contents of residual silicon and carbon in reaction-bonded silicon carbide ceramics (RBSC) has been effectively addressed in a previous study [21]. Several common scientific methods and mechanisms can be beneficially referenced for SiC*_f_*/SiC composites fabricated via the RMI method. However, it is important to note that RMI-SiC*_f_*/SiC composites present more complexities compared to RBSC, primarily due to two factors:

(1) The green body of SiC ceramics can be obtained through simple isostatic pressing or colloidal forming processes, whereas the fabrication of SiC*_f_*/SiC composite preforms involves more intricate methods such as precursor infiltration pyrolysis, chemical vapor infiltration, or slurry impregnation. Additionally, regulating the composition and pore structure of these preforms is more challenging than for SiC ceramic green bodies.
(2) Due to the critical issue of SiC fiber damage at high temperatures, the RMI processing temperature for SiC*_f_*/SiC composites needs to be kept relatively low (≤1600 °C), unlike the higher temperatures (≥1650 °C) feasible for SiC ceramics.

The abovementioned difficulties could lead to the presence of unnecessary free silicon within the SiC ceramic matrix. The melting of residual silicon and its subsequent reactions with the interfacial layer and fibers at elevated temperatures during RMI could adversely affect the performance of the final product. The introduction of pyrolytic carbon (PyC) or BN/SiC interphase layers could partially mitigate the issues related to interfacial reactions [22]. 

Minimizing the amount of residual silicon in the final product is crucial and requires precise control of both the carbon content and the volume/size of the pores in the preforms. Achieving this level of control in the traditional RMI process for preparing SiC*_f_*/C preforms is challenging, particularly because it relies solely on carbon sources. In the RMI process, the pore structure [23,24,25] and size [16,26] of the preform could strongly affect the wettability of liquid Si and the C–Si reaction. The pore structure and size in preforms can be adjusted by synthesizing mesoporous carbons [27] or by changing solid loads [28]. One of the main challenges of the RMI process is designing preforms that yield the least amounts of residual silicon and carbon in the final SiC*_f_*/SiC composite product. In the past, several ideal preforms have been developed to achieve effective RMI. Guo et al. proposed an alternative method to enhance the RMI approach by synthesizing a special porous carbon (C_g_) and incorporating it into porous 2D SiC*_f_*/SiC composites prepared by chemical vapor infiltration (CVI) [29]. This resulted in a two-stage pore structure, leading to more complete RMI and less content and better dispersion of the residual silicon. The SiC*_f_*/SiC composites achieved a high flexural strength of 808.7 ± 10.2 MPa. Chen et al. prepared C*_f_*/B_4_C-C preforms with different pore structures using slurry impregnation and sol impregnation methods [30]. Preforms made by the sol impregnation method exhibited more uniform pore structures and a reduced amount of residual silicon, consequently increasing the composite′s flexural strength from 145 MPa to 192 MPa. Optimized pore structure prefabrication is key to producing high-quality SiC matrix composites via the RMI method. However, these methods involve complex and costly precast production processes, making them less favorable for mass production.

The primary objective of this study was to develop a simple and cost-effective method for the fabrication of high-performance SiC*_f_*/SiC composites based on the RMI approach. The initial step involved creating a fiber preform by impregnating SiC fiber cloth with phenolic resin, followed by laminating and pyrolyzing the assembly. Subsequently, the preform underwent further impregnation with either phenolic resin, silicon carbide slurry, or carbon black slurry. This process was repeated with additional pyrolysis cycles to achieve preforms with tailored compositions and pore structures. These preforms were then subjected to molten silicon infiltration, resulting in the formation of SiC*_f_*/SiC composites. A systematic investigation was conducted to examine the microstructure and properties of the resultant SiC*_f_*/SiC composites. Through comparative analysis, the optimal preformed composition and pore structure for the preparation of SiC*_f_*/SiC composites were identified. Furthermore, the underlying scientific mechanisms governing these observations were elucidated.

## 2. Experimental Procedure

### 2.1. Materials Preparation

**Figure 1:**
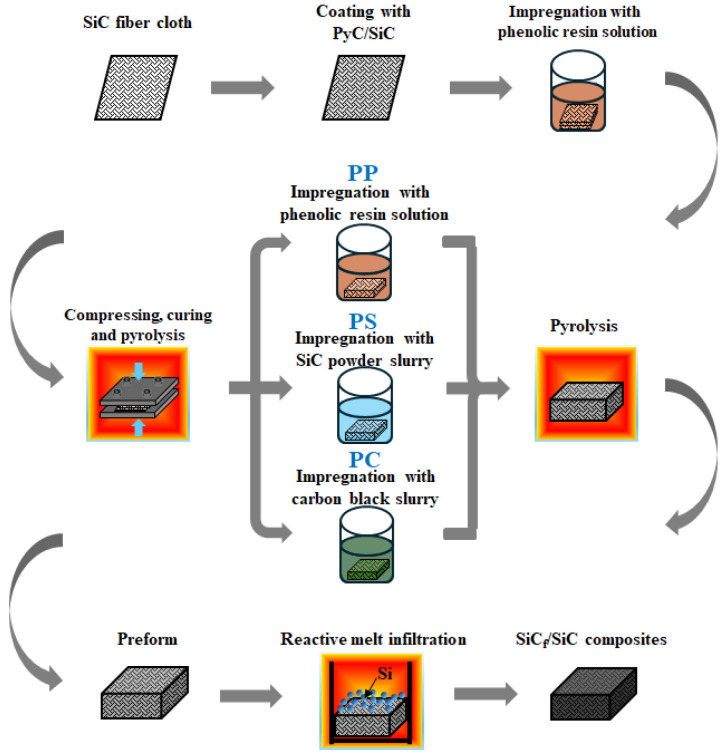
Schematic diagram illustrating the fabrication process of SiC*_f_*/SiC composite samples.

The sample preparation procedures are schematically depicted in Figure 1. Initially, a two-dimensional (2D) satin-woven SiC fiber cloth (Cansas 3303, Fujian Liya New Material Co., Ltd., Quanzhou, China) was coated with PyC via chemical vapor deposition (CVD) technology. Subsequently, a SiC coating was applied using polymer infiltration and pyrolysis (PIP) techniques. The fiber cloth with PyC/SiC coating was then immersed in a 50 wt.% ethanol solution of phenolic resin (BR2123, Henan Borun New Materials Co., Ltd., Zhengzhou, China), placed in an impregnation tank (DN300, Shenyang Weike Vacuum Technology Co., Ltd., Shenyang, China) and subjected to a pressure of 3 kPa for 15 min, followed by an increase to 20 bar for 2 h. Afterwards, 20 stacked sheets of the SiC fiber cloth were compressed using a graphite fixture (Custom specifications, Yuyao Yujun Metal Materials Business Department, Yuyao, China) and subsequently dried and cured at 120 °C for 12 h.

These compacted bodies were then pyrolyzed at 900 °C for 1 h under an argon atmosphere. Following pyrolysis, the compacted bodies were impregnated with phenolic resin, SiC slurry, and carbon black slurry and then pyrolyzed again to obtain three types of preforms: PP, PS, and PC. Specifically, the PP preform was obtained through additional cycles of impregnation with a 50 wt.% phenolic resin solution followed by pyrolysis. The PS preform was prepared using Slurry Infiltration (SI) technology with an aqueous slurry containing 20 vol.% α-SiC powder (D_50_ = 0.5 µm, Qinhuangdao Eno Material Co., Ltd., Qinhuangdao, China) and 2.5 wt.% tetramethylammonium hydroxide (TMAH, Shanghai Aladdin Bio-Chem Technology Co., Ltd., Shanghai, China) as a dispersant based on the mass of α-SiC powder. Similarly, the PC preform was produced using SI technology with an aqueous slurry comprising 30 vol.% carbon black (particle size 20 nm, Qinhuangdao Eno Material Co., Ltd., China) and 2.25 wt.% polyvinylpyrrolidone (PVP, Shanghai Aladdin Bio-Chem Technology Co., Ltd., China) as a dispersant based on the mass of carbon black. For all samples, the second round of impregnation and pyrolysis conditions remained consistent with the first round.

During the subsequent reactive melt infiltration process, the PP, PS, and PC preforms were individually placed in graphite crucibles (Custom specifications, Yuyao Yujun Metal Materials Business Department, Yuyao, China), each topped with an adequate amount of silicon powder (particle size 1–3 mm, purity 99.9999%, Qinhuangdao Eno Material Co., Ltd., Qinhuangdao, China). The samples were then heated to 1600 °C and held under vacuum conditions for 1 h in a graphite furnace (ZGXYS-250, Shenyang Sante Vacuum Technology Co., Ltd., Shenyang, China). This process ultimately yielded SiC*_f_*/SiC composite samples. Prior to the analyses and characterizations, the composite samples were sectioned, surface ground, and polished to a 1 µm finish. 

### 2.2. Characterizations

The densities (ρ) and apparent porosities of the preforms and final SiC*_f_*/SiC composite products were determined using the Archimedes water displacement method. The pore size distributions in the preforms (measuring 10 mm × 10 mm × 10 mm) were analyzed through Mercury Porosimetry (MIP, AutoPore IV 9500, Micromeritics Instrument Co., Ltd., Norcross, GA, USA). The phase compositions of the samples were examined via X-ray diffractometry (XRD: D8 Advance, Bruker Co., Ltd., Karlsruhe, Germany). The microstructures and elemental distributions of the specimens were observed using a scanning electron microscope equipped with an energy dispersive spectrometer (SEM-EDS, Quanta 250 FEG, FEI Co., Ltd., Hillsboro, OR, USA). The three-point bending strengths of bar-shaped samples (measuring 3 mm × 4 mm × 36 mm) were measured using a universal material testing machine (UMT, Z030TE+TEE, ZwickRoell Co., Ltd., Ulm, Germany) with a 20 mm span and a crosshead speed of 0.5 mm/min. The elastic moduli of plate-shaped samples (measuring 100 mm × 30 mm × 10 mm) were evaluated using a non-destructive ultrasonic tester (GrindoSonic system, IET-01, Luoyang Zhuosheng Testing Instrument Co., Ltd., Luoyang, China). The thermal diffusivities (α) and specific heats (C_p_) of square-shaped samples (measuring 10 mm × 10 mm × 2 mm) were estimated using the laser flash method (LFA 467 Hyperflash, NETZSCH-Gerätebau GmbH, Selb, Germany). Thermal conductivity (κ) was calculated based on the relationship κ = ρ⋅C_p_⋅α.

## 3. Results and Discussion

### 3.1. Preforms

The densities and open porosities of the three preform samples are presented in Table 1. The PS preform exhibited the highest density at 1.651 g/cm^3^ and also had the highest apparent porosity of 42.33% among the preforms. The high density of the impregnated SiC powder, at 3.21 g/cm^3^, contributed to the higher density of the PS preform. Unlike nano-sized carbon black, it was more challenging to impregnate submicron-sized SiC particles into the preforms. As a result, the PS preform also had the highest porosity. Furthermore, although both the PP and PC preforms were composed of SiC fiber and C, the PP preform had a lower density of 1.217 g/cm^3^ compared to the PC preform’s 1.428 g/cm^3^. Additionally, the open porosity of the PP preform was 35.17%, which was lower than the PC preform’s 36.39%. This difference is primarily attributed to the fact that phenolic resin tends to generate cracks and closed pores during pyrolysis [30]. As the number of PIP cycles increased, the number of closed pores within the preforms also increased.

**Figure 2:**
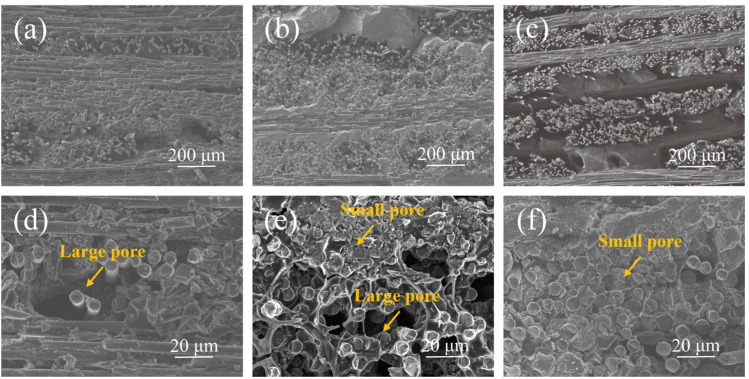
The cross-sectional SEM micrographs of the preforms: low-magnification images of (**a**) PP, (**b**) PS preforms, and (**c**) PC; high-magnification images of (**d**) PP, (**e**) PS, and (**f**) PC preforms.

Figure 2 illustrates the microstructures of the PP, PS and PC preforms. As shown in Figure 2a,d, a significant number of pores were observed on the cross-section of the PP preform, attributed to cracks or large pores formed due to the substantial shrinkage of the matrix during the pyrolysis of the phenolic resin. These pores likely include some closed pores that became exposed during the cutting process for sample preparation. The carbon produced by the pyrolysis of phenolic resin was primarily distributed within the SiC fiber bundles, with minimal carbon content present between the bundles, leading to pore formation. The woven structure of the SiC fiber cloth facilitated the development of closed pores and interlayer cracks between the fiber bundles. Additionally, shrinkage cracks and structural collapse from the pyrolysis of phenolic resin further contributed to the formation of closed pores. Conversely, the carbon matrix within the SiC fiber bundles remained largely intact, featuring a relatively thicker carbon layer, which could hinder subsequent molten silicon infiltration [31].

Figure 2b,e depict that the the interstitial spaces between the fiber bundles of PS preform are filled with a significant amount of SiC powder, effectively reducing porosity. These chemically inert SiC powders did not participate in the reactive melt infiltration process and may have decreased the residual silicon content post RMI. The embedded SiC powders within the fiber bundles create ideal channels for molten silicon infiltration. For the PS preform, the phenolic resin pyrolysis process was conducted only once, resulting in a thinner carbon matrix within the fiber bundles compared to the PP preform. However, large pores were still observed within the fiber bundle. The driving force for molten silicon infiltration into the preform stems from capillary action within these pores; smaller pore sizes indicate stronger capillary forces and enhanced infiltration dynamics [16,23,29]. This mechanism can partially mitigate pore blockage caused by the silicon/carbon reaction. Notably, capillary action within the SiC fiber bundles was more pronounced than between the bundles, ensuring deep penetration of silicon into the preform’s interior and achieving complete infiltration. 

The microstructure of the PC preform is demonstrated in Figure 2c,f. The carbon within the fiber bundles primarily originates from the pyrolysis of phenolic resin, while the interstitial carbon between the bundles mainly comes from impregnated carbon black. The pores within the fiber bundles are extremely small and not visible to the naked eye. Besides, most adjacent SiC fibers are interconnected by a continuous carbon framework formed during the pyrolysis of the phenolic resin. This thermally decomposed carbon framework, along with the impregnated carbon black, would provide an adequate supply of carbon necessary for the subsequent reaction to form SiC during the RMI process. 

**Figure 3:**
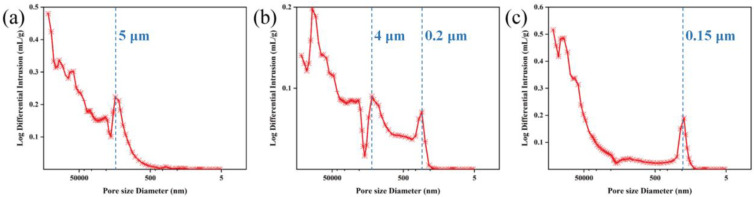
The pore size distributions of the green preforms: (**a**) PP, (**b**) PS, and (**c**) PC.

The pore size distributions of the preforms are illustrated in Figure 3. The pores in the preforms are primarily categorized into micrometer-scale and nanometer-scale pores. Due to the fabrication process involving stacking two-dimensional satin SiC fiber cloth, all preforms inherently contain micrometer-scale pores that are difficult to completely fill. Additionally, all preforms exhibit a multimodal pore size distribution: the PP preform shows peak pore diameters around 5 μm; the PS preform has pores ranging from 200 nm to 4 μm; and the PC preform features pores approximately 150 nm in size. 

The relatively large pores in the PP preform result from the twice-repeated impregnation and pyrolysis of the phenolic resin. For the PS preform, the pyrolysis of the phenolic resin generated large pores, while some of the impregnated SiC powder infiltrated these pores, creating a gradient of pore sizes with smaller pores forming between the fiber bundles. In contrast, for the PC preform, although the pyrolysis of the phenolic resin produced large pores, subsequent infiltration by carbon black effectively filled these pores, resulting in dense packing and leaving only small pores [16].

### 3.2. Composite Materials

**Figure 4:**
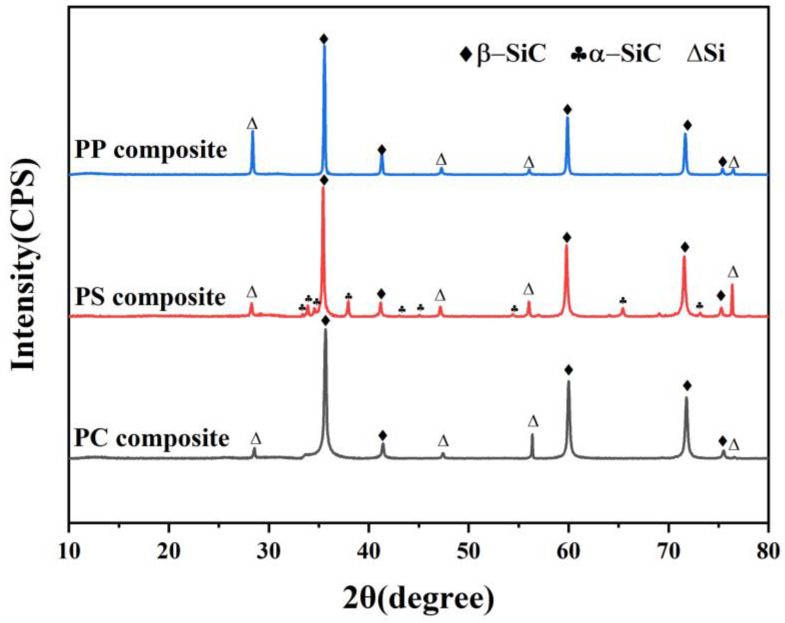
XRD patterns of the final SiC*_f_*/SiC composite samples.

The XRD patterns of the SiC*_f_*/SiC composites are presented in Figure 4. For the PP composite, the predominant phase was identified as β-SiC, with minor peaks corresponding to silicon. Although diffraction peaks of carbon were not identified, its existence could not be excluded due to the amorphous state of carbon black. The formation of β-SiC during the RMI process can be attributed to the following reaction:

Si(*l*) + C(*s*) → β-SiC(*s*) (1)

The PS composite primarily consisted of β-SiC, accompanied by small amounts of both silicon and α-SiC, which were introduced during the slurry infiltration process. In contrast, the PC composite exhibited only diffraction peaks associated with β-SiC and a tiny amount of residual silicon. Among all the samples, the PC composite demonstrated the highest proportion of β-SiC and the lowest amount of residual Si, highlighting its optimal composition for RMI-SiC*_f_*/SiC composites [32].

**Figure 5:**
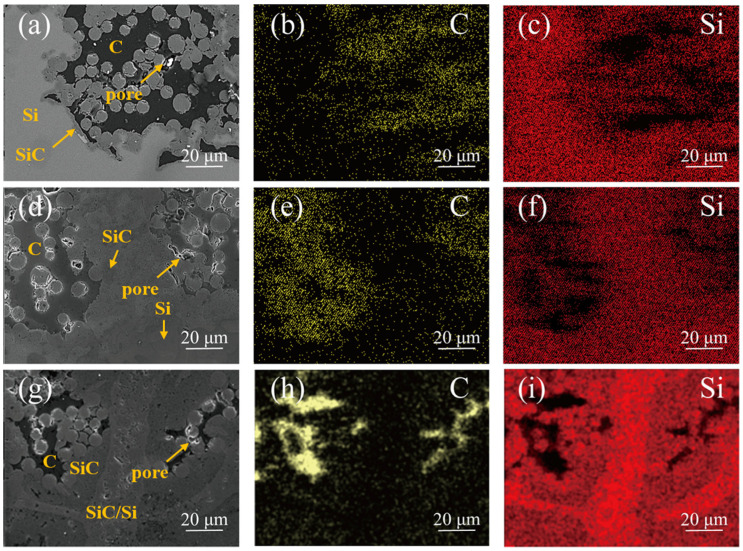
Cross-sectional SEM images and the corresponding EDS mapping of SiC*_f_*/SiC composites: (**a**–**c**) Sample PP, (**d**–**f**) Sample PS, (**g**–**i**) Sample PC.

The microstructures of the SiC*_f_*/SiC composites are illustrated in Figure 5. As shown in Figure 5a, a distinct transition region was observed between the SiC fibers and the matrix in the PP composite sample, primarily composed of β-SiC and Si, as confirmed by previous XRD results. Figure 5a also indicates that in certain areas, the SiC fibers and the matrix were indistinguishable, suggesting the formation of a high-density β-SiC matrix. Figure 5b,c display the distribution of C and Si elements based on EDS analysis, indicating the presence of residual silicon and carbon. Notably, a thin layer of SiC had formed at the C/Si interface, likely inhibiting further reactions between Si and C through spatial hindrance effects. This phenomenon can be attributed to the composition and pore structure of the PP preform. The carbon from phenolic resin pyrolysis formed a continuous C matrix with large pores up to 5 μm within the fiber bundles. The significant contact area for the Si/C reaction led to the formation of a continuous SiC layer, which in turn hindered further interaction between Si and C due to the low solubility of C in SiC and the slow reaction progression [33]. However, the PP preform’s pore structure facilitated the infiltration of molten silicon. The silicon melted, filling the larger pores before entering smaller ones through capillary action. The smaller pores generated higher capillary forces, aiding the entry of the melt into the preform’s interior. This partly offset the adverse effects of pore blockage caused by the Si/C reaction. Although the Si/C reaction occurred early at the interfaces during the RMI process, leading to matrix volume expansion, the molten silicon was unlikely to backflow and continued to infiltrate the preform’s interior [29]. Additionally, the larger pores allowed for an increased amount of residual silicon after the RMI process.

In the case of the PS composite, as presented in Figure 5d, a higher proportion of β-SiC was formed, and there was no distinct boundary between Si and SiC in the matrix, indicating a dispersed distribution. Figure 5e,f show the distribution of C and Si elements. The smaller pore size of the PS preform compared to the PP preform facilitated a more thorough RMI process. Furthermore, the inter-fiber bundles were uniformly filled with SiC powder in the preform, leading to a dispersed distribution of residual Si within the SiC powder in the composite [34]. The presence of residual carbon in the PS composite was primarily due to the similar morphology of carbon produced from the pyrolysis of the phenolic resin, resembling that found in the PP preform. As previously discussed, the peak pore size of the PS preform ranged from 200 nm to 4 μm. Some pores within the powder were smaller than those within the SiC fiber bundles, creating regions where molten Si had difficulty penetrating, resulting in incomplete Si/C reactions. 

In the PC composite, as shown in Figure 5g, over 90% of the observed area displayed an indistinguishable boundary between the fibers and the matrix, indicating a highly ideal microstructure. Figure 5h,i illustrate the distribution of C and Si elements, respectively. The dark regions in Figure 5h predominantly consist of residual carbon. The extensive presence of Si elements in Figure 5i signifies that the Si/C reaction during the RMI process was thorough. The PC preform exhibited the smallest internal pores among the three samples. A smaller pore structure generates higher capillary forces, facilitating a faster and more complete melt infiltration process. The variations in the red’s brightness, indicating differences in Si content, suggest uneven distribution of Si elements between SiC and residual Si regions. Residual carbon may be present in areas encapsulated by SiC, preventing further reaction with Si. The chemical stability of SiC hinders the dissolution of excess carbon into the silicon melt. Additionally, gaps between SiC fiber bundles were filled with SiC, allowing carbon to dissolve in molten silicon rather than forming an inert layer within the fiber bundles. These conditions resulted in a thicker SiC layer compared to the other two samples, contributing to a reduction in residual carbon content. 

Table 2 presents the physical properties of the SiC*_f_*/SiC composite materials. The open porosities of the three samples ranged from 1% to 2%. Ideally, the RMI process filled residual pores with molten silicon, resulting in zero open porosity. However, factors such as silicon shrinkage during solidification and measurement inaccuracies typically led to an open porosity below 2%. The highest density measured was 2.80 g/cm^3^ in the PC composite sample, which was expected to exhibit better comprehensive properties than the other samples. Under ideal circumstances, RMI-derived SiC*_f_*/SiC composites consist of SiC fibers, an interfacial layer, and a SiC ceramic matrix, yielding a theoretical density of approximately 3.21 g/cm^3^. Nevertheless, the practical density fell short of this ideal value due to closed pores formed during the SiC*_f_*/C preform preparation and the RMI process, as well as the presence of unreacted residual carbon and silicon [35]. The PC composite achieved the highest density and lowest porosity, demonstrating that the technical approach of first impregnating with phenolic resin followed by carbon black slurry enables the production of an ideal preformed composite and pore size. These characteristics are crucial prerequisites for subsequent RMI processes aimed at achieving better performance of SiC*_f_*/SiC composites. The elastic modulus of the PC composite reached a maximum value of 181.1 ± 0.1 GPa, which was higher than the 176.3 ± 0.1 GPa of the PS composite and the 145.0 ± 0.1 GPa of the PP composite. The low elastic modulus of the PP composite was attributed to the abundant closed pores within the preform, which inherently possessed zero elastic modulus. For the PS and PP preforms, during the RMI process, the generated SiC was insufficient to fully fill these pores, leaving residual Si to occupy the remaining spaces, along with abundant residual carbon. These pores, along with the residual Si and C, detrimentally reduced the density, strength, and elastic modulus of the composite. The moderate elastic modulus of the PS composite arose from the formation of a relatively thick Si-SiC layer during the fabrication process, which combined with the in situ generated SiC to form a dense SiC structure. The residual Si and C within the PS composite also contributed to a decrease in its elastic modulus. The PC composite preparation route was proved to be more effective in this study.

Table 2 presents the densification results and physical properties of the final SiC*_f_*/SiC composites. The densities of the PP, PS, and PC samples were 2.73 g/cm^3^, 2.75 g/cm^3^, and 2.80 g/cm^3^, respectively, with open porosities ranging from 1% to 2% for all the samples, indicating a high densification degree. Typically, the RMI process fills residual pores with molten silicon, resulting in zero open porosity. However, factors such as silicon shrinkage during solidification typically lead to an open porosity below 2%. The intrinsic density of SiC is 3.21 g/cm^3^. Nevertheless, the actual densities of the composite samples were lower than this intrinsic value, primarily due to the presence of internal pores, residual silicon, and residual carbon [35]. The highest density and lowest porosity of the PC composite demonstrate that the composition and pore size of the PC preform are most favorable for preparing ideal RMI-SiC*_f_*/SiC composites. 

**Figure 6:**
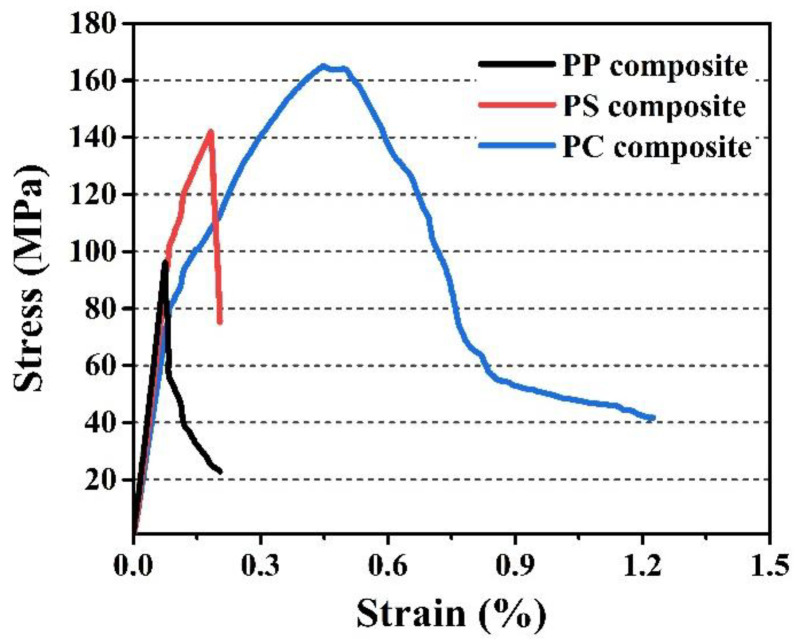
The flexural stress-strain curves of the PP, PS, and PC composite samples.

Figure 6 illustrates the bending stress-strain curves of three SiC*_f_*/SiC composite samples. The curves can be divided into three regions: an initial linear increase, followed by a nonlinear rise, and finally a nonlinear decrease. These features indicate that all composite samples exhibit typical non-brittle failure behavior. However, there were significant differences in the fracture behavior among the three samples. The highest points of the stress-strain curves, representing the flexural strength, showed significant variation among the composite samples. The flexural strengths, ranked from lowest to highest, were as follows: PP, PS, and PC. This order corresponds to the flexural strength values listed in Table 2: 135.7 ± 15.5 MPa for PP, 142.0 ± 7.6 MPa for PS, and 152.4 ± 15.4 MPa for PC. Additionally, the area projected to the *x*-axis by the stress-strain curves represents the work of fracture; a larger projected area indicates better toughness. The order of the projected areas, from smallest to largest, was PP, PS, and PC. 

**Figure 7:**
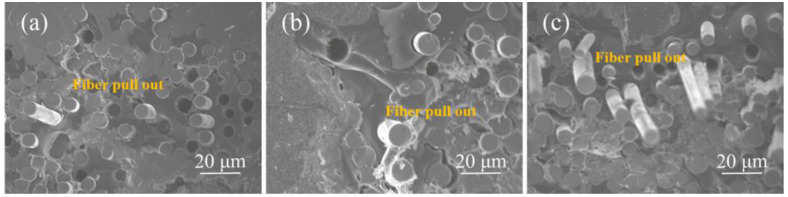
The SEM micrographs of typical fracture surfaces of SiC*_f_*/SiC composite samples: (**a**) PP, (**b**) PS, and (**c**) PC.

To verify these results, typical fracture surfaces of SiC*_f_*/SiC composite samples were observed and compared using SEM, as shown in Figure 7. The fracture surface of the PP sample (Figure 7a) was very planar with only a few short pullout fibers. The number of pullout fibers slightly increased in the PS sample (Figure 7b). In contrast, numerous long pullout fibers were observed in the PC sample (Figure 7c). Fiber pullout is a primary mechanism for toughening, and a higher number of fiber pullouts indicates a more pronounced tough fracture behavior in the SiC*_f_*/SiC composites, consistent with the results presented in Figure 6. Generally, a weaker fiber/matrix interface promotes fiber pullout [36,37]. In this study, the bonding strength at the fiber/matrix interface of the SiC*_f_*/SiC composite samples likely varied due to residual silicon content. Specifically, the PP sample had the highest residual silicon content, possibly allowing molten silicon to penetrate the SiC interlayer and erode the PyC interlayer during the reactive melt infiltration, thereby enhancing the fiber/matrix interface bond and hindering fiber pullout. Conversely, the PC sample had the lowest residual silicon content, preventing molten silicon from breaching the SiC interfacial layer and preserving the PyC interlayer, ensuring a weaker fiber/matrix interface bond and facilitating fiber pullout.

The elastic moduli of the PP, PS, and PC samples were 145.0 ± 0.1 GPa, 176.3 ± 0.1 GPa, and 181.1 ± 0.1 GPa, respectively. Since the elastic moduli of silicon and carbon are significantly lower than that of SiC, a reduction in residual silicon and carbon content effectively enhances the elastic modulus of the SiC*_f_*/SiC composites.

The thermal conductivities of the SiC*_f_*/SiC composites are also presented in Table 2. The thermal conductivity values for the PP, PS, and PC samples were 21.3 W/mK, 24.1 W/mK, and 27.7 W/mK, respectively [38,39,40]. Due to the strong covalent bonding characteristics of Si-C, the primary mechanism of thermal conduction in SiC*_f_*/SiC composites is phonon transport. The differences in thermal conductivity among the samples can be attributed to two main factors:

(1) According to the formula for thermal conductivity, (κ = ρC_p_α), a higher density (ρ) contributes to a higher thermal conductivity. The order of thermal conductivity for the PP, PS, and PC samples aligned with their respective densities.
(2) There were variations in the matrix composition among the different composite samples, specifically significant differences in the contents of residual silicon and carbon, as indicated by XRD and SEM results. As reported in previous studies, the intrinsic thermal conductivities of SiC single crystals (both β- and α-SiC), silicon single crystals, and carbon black are 490 W/mK, 156 W/mK, and 2 W/mK, respectively. Thus, composite samples with higher contents of residual silicon and carbon black exhibited lower thermal conductivity.

Additionally, SiC*_f_*/SiC composite samples were primarily composed of SiC fibers, fiber/matrix interfaces, and ceramic matrix. The ceramic matrix included β-SiC (formed through infiltration reactions), α-SiC (present only in PS samples), residual silicon, residual carbon, and a small amount of porosity. Consequently, SiC*_f_*/SiC composite samples do not exhibit an ideal single-crystal structure but contain numerous defects that cause phonon scattering. These defects include fiber/matrix interfaces, as well as point defects, dislocations, stacking faults, grain boundaries, and pores within the matrix. They disrupt the efficient transfer of phonons, leading to significantly lower thermal conductivity than the intrinsic value of SiC single crystals.

## 4. Conclusions

This study demonstrates that high-performance SiC*_f_*/SiC composites can be achieved using a straightforward and cost-effective RMI method by carefully controlling the preformed composition and pore size. The findings highlight the critical role of preform design in achieving optimized mechanical and thermal properties.

(1) Optimized Preform: The PC preform, prepared through carbon black slurry impregnation, contained an adequate amount of carbon and exhibited an ideal pore size distribution. These characteristics facilitated a more efficient RMI process, resulting in a composite with high density, low porosity, and minimal residual silicon and carbon. This significantly enhanced the mechanical properties of the composite.
(2) Enhanced Properties: Among all the samples tested, the PC composite demonstrated the highest flexural strength (152.4 ± 15.4 MPa), elastic modulus (181.1 ± 0.1 GPa), and thermal conductivity (27.7 W/mK). These exceptional properties are crucial for applications requiring high performance under extreme conditions.
(3) Cost-Effective Approach: The methodology developed in this study provides a simple and cost-effective approach for preparing high-performance SiC*_f_*/SiC composites. By optimizing the preformed composition and pore size, the RMI process was significantly improved, leading to better performance in SiC*_f_*/SiC composites.

## Figures and Tables

**Table 1:** The densities and apparent porosities of the green preforms.

Preform	Density (g/cm^3^)	Open Porosity (%)
PP	1.217	35.17
PS	1.651	42.33
PC	1.428	36.39

**Table 2:** The densification results and physical properties of the SiC*_f_*/SiC composites.

Composites	Bulk Density (g/cm^3^)	Open Porosity (%)	Flexural Strength (MPa)	Elastic Modulus (GPa)	Thermal Conductivity (W/mK)
PP	2.73	1.64	135.7 ± 15.5	145.0 ± 0.1	21.3
PS	2.75	1.96	142.0 ± 7.6	176.3 ± 0.1	24.1
PC	2.80	1.50	152.4 ± 15.4	181.1 ± 0.1	27.7

## Data Availability

The data presented in this study are available on request from the corresponding author.
